# Adipose-Derived Mesenchymal Stromal Cells in Basic Research and Clinical Applications

**DOI:** 10.3390/ijms24043888

**Published:** 2023-02-15

**Authors:** Katarzyna Czerwiec, Małgorzata Zawrzykraj, Milena Deptuła, Aneta Skoniecka, Agata Tymińska, Jacek Zieliński, Adam Kosiński, Michał Pikuła

**Affiliations:** 1 Division of Clinical Anatomy, Department of Anatomy, Faculty of Medicine, Medical University of Gdansk, 80-211 Gdańsk, Poland; 2 Laboratory of Tissue Engineering and Regenerative Medicine, Division of Embryology, Department of Anatomy, Faculty of Medicine, Medical University of Gdansk, 80-211 Gdańsk, Poland; 3 Department of Oncologic Surgery, Medical University of Gdansk, 80-214 Gdańsk, Poland

**Keywords:** AD-MSCs, fat tissue, stem cells, regenerative medicine

## Abstract

Adipose-derived mesenchymal stromal cells (AD-MSCs) have been extensively studied in recent years. Their attractiveness is due to the ease of obtaining clinical material (fat tissue, lipoaspirate) and the relatively large number of AD-MSCs present in adipose tissue. In addition, AD-MSCs possess a high regenerative potential and immunomodulatory activities. Therefore, AD-MSCs have great potential in stem cell-based therapies in wound healing as well as in orthopedic, cardiovascular, or autoimmune diseases. There are many ongoing clinical trials on AD-MSC and in many cases their effectiveness has been proven. In this article, we present current knowledge about AD-MSCs based on our experience and other authors. We also demonstrate the application of AD-MSCs in selected pre-clinical models and clinical studies. Adipose-derived stromal cells can also be the pillar of the next generation of stem cells that will be chemically or genetically modified. Despite much research on these cells, there are still important and interesting areas to explore.

## 1. Introduction

Characterization of Adipose-Derived Mesenchymal Stromal Cells

Nowadays, adipose-derived mesenchymal stromal cells (AD-MSCs), also known as adipose-derived stem cells (ASCs), enjoy great interest among researchers. These cells have great potential in the development of regenerative medicine, wound healing, and tissue reconstruction [1,2]. AD-MSCs represent a multipotent population of cells [3,4,5]. Their main source, as the name says, is adipose tissue. In vitro, AD-MSCs show the typical mesenchymal cell characteristic among others, and the cells adhere to the plastic cell culture flasks under standard culture conditions [3,4,6]. They express surface antigens such as CD73, CD90, and CD105 without expression of CD45, CD19, CD14, CD11b, and HLA-DR [3]. AD-MSCs are also capable of differentiation into adipogenic, osteogenic, and chondrogenic lineages under appropriate in vitro conditions. They also possess high paracrine activities; they produce a large number of cytokines and growth factors. AD-MSCs also interact with many kinds of cells including cells of the immune system. AD-MSCs belong to mesenchymal stem cells which are also called medicinal signaling cells (MSCs) [7].

Adipose tissue is harvested during surgery or liposuction and has usually been treated as medical waste [1]. This is less invasive compared to isolating stem cells from the bone marrow (BM-MSCs). There are no ethical contraindications for collecting research material as in the case of embryonic stem cells [8]. The method of isolating stem cells from adipose tissue is relatively simple to obtain. This particular cell type shows immunosuppressive, anti-inflammatory, and angiogenic properties through the release of soluble mediators in a paracrine way. They have a limited replication life, resulting in a reduced risk of malignancy compared to embryonic stem cells and induced pluripotent stem cells (iPSCs) [7]. Both autologous and allogeneic transplants are used in clinical trials with the use of AD-MSCs. Another advantage of AD-MSCs is the low risk of immune rejection in the allograft approach. However, when deciding to use AD-MSCs, several disadvantages must be considered. This particular population of cells has a limited capacity for self-renewal. Compared to BMSCs, they have a longer replication lifespan. It is also known that decreasing proliferation and differentiation potential of AD-MSCs occurs with increasing age, body mass index (BMI), diabetes mellitus, or exposure to radiotherapy [9,10].

Obtaining a large amount of adipose tissue is a key process to have enough cells for a variety of research in an in vitro culture. The collection of the desired biological material necessary for the isolation of AD-MSCs cells may be performed by lipoaspirate collection, liposuction, and excision. In 2001, Zuk et al. [11] proposed a method of isolating AD-MSCs, which later became popular among other researchers with some improvements as well. In the beginning, the collected excess of adipose tissue is washed several times in a phosphate-buffered saline (PBS) solution. Later, the biological material is digested at 37 °C with 0.075% collagenase type I. Next, the enzyme activity is usually neutralized with DMEM medium, which contains 10% fetal bovine serum (FBS). To obtain a high-density stromal vascular fraction (SVF) pellet the contents of the tube are centrifuged at 1200× *g* for 10 min. Then the fraction is suspended in 160 mM NH_4_Cl and incubated for 10 min at room temperature. This step is to lyse the red blood cells. Next, the pellets are cleaned by repeated centrifugation in a neutralizing medium, and then placed in a plastic culture flask with an appropriate medium. Our own experience confirms that AD-MSCs retain their phenotype until passage 6 [2].

The SVF derived from adipose tissue is a heterogenous population of cells such as mesenchymal progenitor stem cells, preadipocytes, endothelial cells, pericytes, T cells, and M2 macrophages [12,13,14]. Some researchers try to use the SVF in regenerative medicine or plastic surgery [12]. The advantage of using SVF is primarily the ease of obtaining the preparation and no need to culture cells in vitro. However, one of the problems is the risk of unexpected cell differentiation and relatively high immunogenicity of SVF (in the case of allotransplantation). In addition, the stromal fraction contains a heterogeneous population of cells, so the mechanisms of action in the patient may be diverse. It is worth noticing that the SVF fraction as well as AD-MSCs represents paracrine mechanisms of action and the capability to differentiate into other cells [1,5,12].

The aim of this review was to summarize current knowledge about the characteristics, biology, and clinical potential of AD-MSCs. Additionally, based on our experience, we wanted to emphasize how these cells look in an in vitro cell culture, and behave after differentiation into particular cell lines.

## 2. Flow Cytometry Characteristic

Flow cytometry is a very useful tool for stem cell analysis. This technique allows, in particular, to confirm the phenotype of cells, assess the level of expression of markers and analyze cell viability [15]. The International Federation of Adipose Therapeutics (IFATS) and the International Society of Cellular Therapy (ISCT) provided guidance for the scientific community on cytometric analysis of AD-MSCs [16]. They propose the use of multi-color analyses. The authors recommended the use of viability dye and surface antigens to characterize the MSCs such as CD73, and CD90. They suggest putting in CD13 as an alternative or supplement to CD105, because the expression level is often more stable and higher. The second reason is that the commercial antibodies targeting this antigen show higher specificity and signal intensity [16]. As for AD-MSCs, they should be negative (<2%) for markers such as CD11b and CD45 and positive for stromal markers—CD13, CD73, and CD90 (Table 1). To distinguish AD-MSC from other MSC, the authors suggest the use of two other markers CD36 (GPIIIb) and CD106 (VCAM-1). The authors claim that the activity of CD34 depends on the culture condition. This antigen is expressed during the early phase of culture. After 8–12 population doublings expression of CD34 decreases with consecutive passages of cells [16].

## 3. Immunomodulatory Effects of AD-MSCs

Transplanted AD-MSCs have the ability to the migrate to inflammation areas and stimulate strong immunomodulatory and anti-inflammatory effects, which is taking place through cell–cell contact—between components of the immune system and AD-MSCs or soluble factors [17]. AD-MSCs can react as modulators of the host response, because they can demonstrate a greater in vitro immunomodulatory ability than BMSCs derived from donors of similar age [18]. Immune cell functions are regulated by contact-dependent mechanisms that involve cell-to-cell contact and effects expressed by various soluble factors and the production of cytokines. Scientists showed in their research a correlation that increased AD-MSCs immunomodulation is associated with greater cytokine production. AD-MSCs, by preventing the proliferation of B lymphocytes and their differentiation into plasma cells, inhibit the production of antibodies. It has been provided that the immunosuppressive ability of AD-MSCs is dose and cell-passage dependent [16]. In order to prepare the appropriate number of cells for in vitro tests, cell passage is performed. After several passages, AD-MSCs may lose their immunosuppressive character. It has been reported that for coculture AD-MSCs and B cells, the former cells have an inhibitory effect on the chemotactic properties of the latter, by downregulating chemokine receptors on B cells especially CXCR4, CXCR5, which are receptors for B lymphocyte chemokines; their function is disregarded by the inhibitory effect of cells [16]. Mun et al. [19] studied and compared the phenotypes and cytokine expressions from early to late passages from AD-MSCs and BM-MSCs. Researchers proved that both types of cells were not significantly changed and original cell morphology and population doubling time were not significantly different. They also proved that MSCs are adequate for long-term culture in vitro without losing their essential stem cell features. Mun et al. [19] checked the expression of genes such as TSG-6, galectin-1 and -3, and HLA-G from early to late passages. A significant decrease in TSG-6 and HLA- G expression was observed in both types of cells. TSG-6 and HLA- G are connected via the immune system; TSG-6 is expressed by cells in response to pro-inflammatory cytokines, whereas HLA-G exerts an anti-inflammatory effect. Expressions of gelactin-1 and -3 were significantly increased in both types of cells from early to late passages. Galectins have a broad variety of functions including cell–cell interactions, cell–matrix adhesion, and transmembrane signaling [19].

Fiori et al. studied the immunomodulatory potential of AD-MSCs on CD4+ T cells [20], addressing potential cell-contact dependency in relation to T cell receptor stimulation of whole human peripheral blood mononuclear cells (PBMC). They provided the strong AD-MSC inhibitory potential on CD4+ cell proliferation upon PBMC stimulation. The authors also showed that CD25 expression is increased by the stimulation of PBMC. CD4+ CD25+ cell proliferation occurs in cocultures even though AD-MSCs inhibit overall proliferation [20].

## 4. Differentiating Potential

AD-MSCs have a natural tendency to differentiate into adipocytes in vitro. However, under the influence of appropriate growth factors and microenvironmental conditions, they relatively easily differentiate into chondrocytes and osteocytes. The differentiation potential of these cells, however, depends on many factors, including the age and sex of the donor, and the anatomical location of the fat [1,5]. Kawagishi-Hotta et al. [21] analyzed age-related changes in the numbers and potential of human AD-MSCs. They showed that adipogenic potentials get smaller with age. Proliferation and chondrogenic and osteogenic potentials are not correlated with donor age. Moreover, no correlation was observed between BMI and the yield of SVF cells from adipose tissue. The authors Kawagishi-Hotta M et al. indicated [21] that individual differences in AD-MSC changed with aging; the individual differences became significant after the patients reached 60 years of age. AD-MSC from male donors represented a significant increase in individual differences in AD-MSC after the age of 80; for female donors was the age of 60. They also demonstrated the expression levels of MSC surface makers and the undifferentiated cell marker in AD-MSC from the young and elderly donor groups. They noticed that in the young group, the expression level of CD105 was different depending on the proliferation potential level. In the elderly group, there were differences in the expression of CD73 and CD74, depending on their adipogenic and osteogenic potential.

Growth factors have a strong effect on both proliferation and differentiation of stem cells. They are therefore also of great therapeutic importance, especially in regenerative medicine. TGF-beta (transforming growth factor beta) and BMPs (bone morphogenetic proteins) signaling regulate bone formation during mammalian development and versatile functions in the body. Grotheer et al. [22] demonstrated that TGF-beta signaling inhibited the osteogenic differentiation of AD-MSCs. BMP-2 supplementation significantly inhibited their osteogenic differentiation potential at passages 3 and 10. Administration of dorsomorphin during osteogenic had a short-term inhibitory effect on day 14 at passage 3 [22]. Inhibition of TGF-beta signaling essential started the osteogenic differentiation potential to similar levels at passages 3 and 10 [20]. Grotheer et al. showed that low expression of CD44 was significant in AD-MSCs. CD44 is involved in cell–cell interactions, migration, and cell adhesion. The osteogenic differentiation potential at passage 10 barely decreased compared to passage 3 [22]. The same thing happened with the osteogenic differentiation pattern. BMP-2 supplementation significantly inhibited osteogenic differentiation. The inhibition of TGF-beta signaling essentially accelerated the osteogenic differentiation potential [22].

Mohiuddin et al. [23] analyzed the effect of AD-MSCs and decellularized adipose tissue-derived (DAT) hydrogel interaction on AD-MSCs differentiation and DAT hydrogel microstructure. They seeded AD-MSCs on DAT hydrogel and cultured them in stromal and adipogenic or osteogenic media for 14–28 days. Adipogenic differentiation showed the upregulation of adipogenic markers genes and an accumulation of oil droplets in cells [23]. Osteogenic differentiation exhibited the osteogenic markers genes and mineral deposition in the DAT hydrogel [23]. DAT hydrogel matrix revealed that AD-MSCs seeding and differentiation altered the diameter and arrangement of fibers in the matrix. They also demonstrated that attachment of AD-MSCs and differentiation along osteogenic and adipogenic lineages remodels the microstructure of DAT hydrogel [23].

Adipose-derived stromal cells are stem cell populations that have easy acquisition and multiple differentiation potentials making AD-MSCs attractive for tissue engineering and cell therapy as an ideal stem cell source for research.

Zampar et al. analyzed AD-MSCs from different donor areas: upper abdomen, lower abdomen, tights, dorsum, and flanks [24]. They investigated differences in the AD-MSCs by analyzing the action of the supernatant produced by AD-MSCs from different body areas on fibroblast migration, and differences in the secretome present in the supernatant produced by these cells. The biological material was collected by liposuction. Their work showed that the dorsum provided the highest concentration of AD-MSCs than the other areas of the body [24]. Analysis of the secretomes showed a significant difference in VEGF (vascular endothelial growth factor) concentrations, which were lower in the control group. VEGF has a crucial role in tissue healing closely linked with migration and proliferation in healing-related cells [24].

## 5. Adipose-Derived Mesenchymal Stromal Cells (AD-MSCs) Versus Bone-Marrow-Derived Mesenchymal Stem Cells (BM-MSCs)

Mesenchymal stromal stem cells were first discovered by Friedenstein’s team, who observed non-hematopoietic, non-phagocytic cells, similar in shape to fibroblasts. The newly discovered cells were isolated from rat bone marrow [25,26,27]. Thanks to many publications and reports, the state of knowledge about stem cells from the bone marrow is very large. In 2001, researchers’ attention was drawn to the report of Zuk, who was the first to characterize AD-MSCs [11,28,29]. Obtaining fat tissue and then stem cells is generally a simpler and safer procedure compared to the mechanical isolation of mesenchymal cells from the bone marrow. Adipose tissue or lipoaspirate is often medical waste that can then be used for research or clinical applications [30,31,32]. It is believed that different isolation techniques may influence the characteristics of stem cells [33]. Some authors claim that human AD-MSCs in long-term cultures are morphologically more stable than BM-MSCs [34]. Compared to mesenchymal stem cells isolated from the bone marrow, they show a higher proliferative capacity and a lower senescence rate. First of all, AD-MSCs retain a higher differentiating potential. It is well known that relatively few BM-MSCs are obtained from the bone marrow stroma. The frames range from 0.001–0.01% of all bone marrow nucleated cells. On the other hand, the number of isolated cells from 1 g of adipose tissue is 1000 times higher than from 1 g of bone marrow [35]. This difference may be crucial and explain why AD-MSCs have become so popular for choice in basic research and clinical applications [32,36,37,38].

It is well known that the immunophenotype of AD-MSCs and BM-MSCs is mostly identical, but there are also minor differences between the two cell types (Table 2 and Table 3). AD-MSCs are known to express the surface marker CD34 when these cells are freshly isolated and the expression of this marker decreases with subsequent passages. BM-MSCs and MSCs from other sources do not express CD34 [16,39,40]. CD49d known as integrin α-4 is highly expressed by AD-MSCs and not by BMSCs, and CD106 known as vascular cell adhesion molecule-1 is highly expressed by BMSCs and not by AD-MSCs [41]. As for further differences in the immunophenotype of AD-MSCs and BM-MSCs, the surface markers CD45 (protein tyrosine phosphatase receptor type), CD133 (prominin-1), and CD144 (vascular endothelial cadherin) are expressed in BM-MSCs and not in AD-MSCs. A slight difference can be distinguished as regards the marker Stro-1; BM-MSCs show its higher expression than AD-MSCs. In terms of similarities, AD-MSC and BM-MSC show the same amount of expression for CD13 (alanyl aminopeptidase, membrane), CD29 (integrin subunit beta), CD73 (5′-nucleotidase ecto), CD90 (Thy-1 cell surface antigen), CD105 (endoglin), and HLA-DR (major histocompatibility complex) [40,42]. It should be emphasized that the immunophenotype depends on the passage in which the cells are found [43].

Both types of mesenchymal stem cells have the potential for differentiation [44]. Studies have shown that adipogenic differentiation is much stronger in AD-MSCs than in BM-MSCs. There are also studies that have shown that the osteogenic potential is greater for BMSCs than for AD-MSCs. Gender seems to play an important role in osteogenic differentiation in AD-MSCs. AD-MSCs isolated from males are characterized by faster and more effective differentiation than AD-MSCs isolated from females. It is also claimed that the osteogenic potential of AD-MSCs isolated from women decreases with age. There are also studies that have shown that the chondrogenic potential is greater with BM-MSCs than with AD-MSCs [33,39].

Zhou et al. [45] compared AD-MSCs and BMSCs using single-cell RNA sequencing. Researchers showed that AD-MSCs showed lower transcriptomic diversity than BM-MSCs. The team reports that AD-MSCs are less dependent on mitochondrial respiration for energy production. In addition, AD-MSCs showed lower expression of human leukocyte antigen class I antigen and higher immunosuppression capacity than the BM-MSC population [45].

**Table 2:** Comparison of marker expression in AD-MSCs and BM-MSCs.

S	AD-MSCs	BM-MSCs	References
**CD13**	++	++	[16]
**CD 14**			[39,40,43,46]
**CD29**	+	+	[42]
**CD 34**	++	−	[39,40,46]
**CD 44**	+		[42]
**CD 45**	−	+	[16]
**CD 49d**	++	+	[39]
**CD 73**	+++	+++	[39]
**CD90**	+++	+++	[39]
**CD105**	+++	+++	[39,42]
**CD106**	±	++	[16,42]
**CD 133**	−	+	[39,40,43,46]
**CD 144**	−	+	[39,40,43,46]
**HLA-DR**	−	−	[46]
**Stro-1**	+	++	[39]

+++ ≥70%, ++ 30–70%, ± 2–30%, − ≤2%.

**Table 3:** Phenotypic features of mesenchymal stem cells.

	AD-MSCs	BM-MSCs	References
**Proliferation**	Higher rate of proliferation; Late differentiation.	Lower rate of proliferation; Earlier maturation.	[39]
**Adipogenic differentiation capacity**	Intensified formation of lipid vacuoles.	Weaker formation of lipid vacuoles.	[39,42]
**Osteogenic differentiation capacity**	Slowed process of calcium deposits.	More intensive deposition of calcium deposits; mineralization.	[42,46]
**Chondrogenic differentiation capacity**	Lower chondrogenic capacity.	Higher chondrogenic capacity.	[39]

## 6. Metabolism of Adipocytes and AD-MSCs

Adipose tissue is known as energy storage. It is also responsible for thermal isolation as well as the storage and production of hormones, proteins, and cytokines. The basic building blocks of adipose tissue are adipocytes, which can be usually divided into white and brown adipocytes [47,48]. White adipocytes form a cluster of cells with unilocular lipid droplet, which takes up almost the entire volume of the cells. They store energy in the form of triacylglycerol. Clusters of white adipocytes form white adipose tissue (WAT). Within the WAT, we can distinguish between subcutaneous and visceral adipose tissue. Subcutaneous adipose tissue is related to the dermis layer of the skin, whereas visceral adipose tissue is bound around organs inside the intra-abdominal cavity. Subcutaneous adipose tissue works as a protection of internal organs against mechanical damage; visceral adipose tissue is characterized by higher lipolytic responses. It also releases free fatty acids. Visceral fat, which secretes free fatty acids, and its presence in the liver area may contribute to its negative impact and the development of metabolic diseases, including insulin resistance, atherosclerosis, or arterial hypertension [47,48]. Brown adipose tissue acts as an endocrine organ. It is built by brown adipocytes, which include many lipid droplets and numerous mitochondria. Brown adipocytes contain multiple connections of blood vessels. Mitochondria in brown adipose tissue express the mitochondrial protein uncoupling protein 1 (UCP1). Thanks to this protein, brown adipocytes participate in the production of heat from stored energy. Brown adipose tissue is regulated by the sympathetic nervous system. Gap junctions increase the effect of the noradrenergic stimulus, which causes activation of brown adipocytes. Thanks to this, it is possible to induce mitochondrogenesis, lipolysis, and UCP1 synthesis [47,48]. Beige adipocytes are UPC1-positive adipocytes, which we can find in white adipose tissue. They usually behave like white adipose tissue, but differ in that they behave like brown adipose tissue under certain factors, such as exposure to cold or administration of catecholamines [47,48].

Diabetes mellitus belongs to chronic metabolic disease, which is associated with inappropriate insulin secretion and disturbances in the hormone signaling pathway [49]. Insulin resistance is the primary cause of most type 2 diabetes. Osteogenesis is closely related to the insulin signaling pathway. Osteoblasts, under the influence of the activation of insulin receptors, stimulate proliferation and activate the synthesis of collagen and osteocalcin. Skubis-Sikora et al. [49] indicated that non-insulin-dependent diabetes mellitus decreased the proliferation activity of AD-MSCs and changed the phenotypic characteristics. What is noteworthy in the studies by these scientists is that AD-MSC differentiation into osteoblasts was stronger in patients with type 2 diabetes. Changes in the metabolism of people with diabetes can cause disruptions in the reconstruction process—abnormal microarchitecture, abnormal new bone formation, and changes in the composition of bone tissue, which is softer in diabetics compared to healthy people. In patients, the expression of genes responsible for the formation of osteoblasts leads to an increased expression of pro-inflammatory cytokines [49]. It is noteworthy to investigate the effects of metformin, which may also affect multiple molecular pathways.

Qiang Li et al. [50] showed that AD-MSC in high glucose stress indicates induction of autophagy and apoptosis and an essential increase in intracellular reactive oxygen species (ROS) levels. They indicated that the JNK signaling pathway was involved in high glucose-induced autophagy [50]. All their data demonstrated that targeting autophagy may be a clue for possible uses of AD-MSCs in cell therapies, especially for diabetic patients.

Obesity leads to disturbances in the metabolic pathways of the organism. The ubiquitous availability of food, especially in highly developed countries, and the lack of physical effort leads to a measurable accumulation of nutrients. Obesity not only leads to metabolic dysfunctions, but also to aging processes, and the increased risk of other health problems. Nitya Shree et al. [51] injected human AD-MSC in various forms in high-fat-diet-induced C57BL/6 mice. They observed that the animals demonstrated a decrease in insulin resistance. Administration of cell suspension improved glucose tolerance and reduced fatty infiltration in the liver. All the treatments led to a reduction in proinflammatory IL-6 [51]. Obesity combined with metabolism dysfunctions with frequent occurrence of inflammation can reduce the proper inflammatory response. It also affects the ability to regenerate muscles. These phenomena are apparent in the course of diabetes. Conley et al. [52] proved that obesity causes early aging in AD-MSC. Damage to cells caused by obesity can damage their repair system. This may contribute to the difficulty of autologous transplants in obese people.

Mengzhu Lv et al. [53] showed the use of AD-MSC cells that could be significant in the future in terms of aging-related diseases. They proved that AD-MSC can influence metabolic homeostasis through mitophagy, which can delay the aging process. Their research was carried out in a cell and mouse model. AD-MSCs caused mitochondrial membrane protein markers and cytoplasmic proteins to decrease, demonstrating that the number of mitochondria in the cell model had decreased [53]. The effect of AD-MSCs reduced the production of mitochondrial ROS. In the animal model, the administration of AD-MSC delayed aging in mice with mitochondrial dysfunction. Western blot analyses showed that the number of tissue mitochondria in the research group decreased and autophagy increased [53].

## 7. Therapeutic Potential of Exosomes

Exosomes are a subtype of the extracellular vesicles (EVs), in size 30–200 nm, which contain diverse biological molecules (functional proteins, nucleic acids, and lipids) for intercellular communication, and multiple biological processes such as cellular proliferation, differentiation, or apoptosis [54]. Exosomes are produced by all cell types and display similar properties as their parental cells, including their molecular composition and function, which is why exosomes seem to be an opportunity for the development of cell-free therapeutics [55,56,57].

Mesenchymal stem cells` exosomes are a potential therapeutic platform for a number of human diseases in regenerative medicine, e.g., liver fibrosis, lung disorders, osteoarthritis, colitis, myocardial injury, spinal cord injury, and retinal injury [58,59]. Exosomes originating from adipose-tissue-derived MSCs, in liver fibrosis, up-regulate miR-122, which reduces collagen maturation and extracellular matrix synthesis [60].

Recently, researchers have pointed out that exosomes derived from AD-MSCs have anticancer activity on the immunocompetent syngeneic mouse model of breast cancer. In vitro assays indicated that exosomes with a lower level of CD90 expression (CD90^low^ were obtained by LPS stimulation on CD90^high^) showed stronger inhibition in tumor-cell proliferation and weaker migration compared to exosomes with CD90^high^. AD-MSC-exosomes CD90^low^ by addition anti-oncogenic miRNA-16-5 p were used as drug carriers to treat breast cancer in tumor-bearing mice, and caused the best results by robustly increased-level tumor-cell apoptosis, slowed tumor growth, and decreased tumor mass [61].

There were also attempts to use the allogeneic AD-MSC-exosomes to treat kidney disease in mice. Unfortunately, AD-MSC-exosomes result in the exacerbation of the progression to end-stage kidney disease [62].

AD-MSC-exosomes also seem to have the potential for cell-free therapeutic in dermatology. Initial research with their use in atopic dermatitis treatment showed symptom amelioration by a reduced expression of mRNA of cytokines (IL-4, IL-23, IL-31, and TNF-α) in an in vivo mouse model [63]. Similar results (decreased levels of IL-5, IL-13, TNF-α, IFN-γ, IL-17, and TSLP) were visible after subcutaneous injection of AD-MSC-exosomes in an oxazolone-induced dermatitis model [64].

AD-MSCs are known as a stimulation factor in the process of tissue regeneration, so AD-MSC-exosomes are supposed to be a novel method for scarless wound repair. Intravenous injection of AD-MSC-exosomes in a mouse skin incision model showed enhanced extracellular matrix (ECM) reconstruction through a change in the ratio of collagens or TGF factors [65,66]. AD-MSC-nano-vesicles increase collagen I and III production, especially at an early stage of wound healing. Moreover, other MSC-derived extracellular vesicles show properties preventing scar formation [67].

Exosomes derived from stem cells are currently being explored mainly because of their protective and immunomodulatory properties [55]. Exosomal products have not been approved by the FDA. Low productivity of exosomes, collection of high-quality and uniform exosomes, purity properties, standardization of storage conditions, or delivery of exosomes into target tissue/cell are critical issues [68].

## 8. Clinical Applications

In recent years, many studies have focused on using the regenerative potential of AD-MSCs in the treatment of various diseases, including autoimmune [69,70], neurodegenerative [71,72], orthopedic disorders [73,74], and skin damage (Table 4) [9,75]. At the same time, stem cells are utilized to regard complications such as fistula in Crohn’s disease (CD). The fistula in CD is due to a defect in the epithelium and is manifested by the formation of a link between organs. Most often, the fistula appears around the anus, less often in the intestines. Treatment methods are mainly based on antibiotic therapy or the use of immunosuppression. However, procedures are limited [76]. In response to the lack of effective therapy, many clinical trials with the use of AD-MSCs have been conducted [NCT01011244, NCT00992485, NCT01372969, NCT00999115, NCT01314079]. One of them resulted in the launch of Darvadstrocel (Alofisel^®®^), the first AD-MSC-based medical product accepted in the European Union. Alofisel^®®^ contains 120 million allogeneic human AD-MSCs introduced into the lesion. This therapy results in remission of more than 52 weeks in further than half of the patients without serious unpropitious effects [77].

According to clinicaltrials.gov, 209 studies about adipose-derived stroma have been reported, 62 of which have completed status reported (search: adipose derived stem cell, https://clinicaltrials.gov/, accessed on 10 January 2021). Researchers used both origins of stem cells: allogeneic and autologous. Clinical trials have focused on several diseases such as skin disorders, including burns, scars, and chronic wounds. Furthermore, AD-MSCs have been used in autoimmune ailments such as rheumatoid arthritis or systemic scleroderma, which is a chronic disease of connective tissue. At the same time, studies are based on orthopedic defects resulting from trauma, tibia fracture, lateral epicondylitis, or osteoarthritis of the knee joint. Clinical therapies are also applied in the case of cardiovascular diseases, including myocardial infarction, coronary atherosclerosis, coronary artery disease, or ischemic heart disease. Among the neurodegenerative ailments, researchers concentrated on the above-mentioned fistula in the course of Crohn’s disease. Due to the very high biological activity of adipose tissue stem cells, further new clinical trials should be expected. Especially interesting seems to be the preclinical results of research on modified cells, which have a very specific biological effect (the next-generation stem cells) [78]. Various clinical trials are presented in Table 4.

## 9. Safety Issues and Side Effects of AD-MSCs Application

Stem cells have a very high regenerative potential and biological activity. However, due to the very diverse mechanisms of action and high donor-to-donor variability, their clinical effects are not fully predictable. This may be associated with some side effects and reduced clinical efficacy in some clinical applications. Although the efficacy of cellular therapies was confirmed in many cases, there is still a lot of work to be completed regarding their exact mechanism of action, dosage, treatment schedule, and route of administration. All these issues can affect the safety of cellular-based products and should be considered when planning treatment for the patient [79,80].

Safety issues still constitute a barrier to the translation of MSCs-based therapies to everyday clinical practice. There are some concerns about the tumorigenic potential of cultured cells, their immunogenicity, and patients’ susceptibility to infections because of their immunomodulatory effect. In every case, safety and possible risk to the patients should be weighed against the potential benefits of the treatment [81,82].

What is worth noting, is that MSCs were reported not to express the MHC class, which means that they can be used as allogenic or autologous therapy with a minimal risk of immune rejection [83]. However, further analyses of MSCs’ immunobiology showed that they are able to induce an innate and humoral immune response and this issue has been indicated as a barrier to their clinical efficacy. On the other hand, results of pre-clinical and clinical trials of allogenic MSCs indicate that their efficacy is not dependent on the host immune response, probably due to MSCs activity which occurs before the recognition by the host immune system or mechanism of action which includes host immune system [84].

Analysis of the literature data shows reports of adverse events after the application of MSCs and AD-MSCs. For example, the injection of AD-MSCs around the eye during a cosmetic procedure led to the formation of bone-like tissue in the eyelid [85]. Moreover, severe visual loss after intravitreal injections of autologous AD-MSC has been reported in three patients with age-related macular degeneration (AMD). However, it is important to note that these side effects occurred not in the treatment planned in the registered clinical trial. Unfortunately, some stem cell clinics apply cell-therapy products with no proof of efficacy and solid pre-clinical evaluation. What is more, these procedures are often founded by the patient and they lack oversight of regulatory bodies such as the FDA. That is why, when analyzing the side effects of cell therapies, it is important to distinguish clinical trials with solid pre-clinical evidence and scientific design and such attempts performed without a strong scientific base [86].

In the literature, there are also case reports of thrombotic events after intravenous administration of MSCs. For example, a 41-year-old man who received autologous AD-MSCs three times for herniated cervical discs was reported to have chest pain and multiple pulmonary artery embolisms and infarction in the right lung one month after the last dose. Moreover, his parents, who received five doses of autologous MSCs intravenously for knee osteoarthritis, were diagnosed to have multiple pulmonary embolisms in chest CT but without any symptoms [87]. As patients who can benefit from MSC therapy, such as diabetics or people with cancer or inflammatory disorders, are at higher risk of thrombotic events, it is important to carefully monitor MSCs’ pro-thrombotic activity [88].

On the other hand, meta-analyses and systemic reviews of clinical trials confirm the safety of MSC-based therapies. In 2012, Lalu et al. [82] prepared a systemic review and meta-analysis of the safety of MSCs therapy. They searched MEDLINE, EMBASE, and the Cochrane Central Register of Controlled Trials to identify clinical trials of intravascular MSCs administration in adult and mixed (adult and pediatric) populations. Studies utilizing non-intravascular MSCs, differentiated cells, or co-administration of MSCs with other cells or treatment, were not taken into account. They included a total of 1012 participants, which included healthy volunteers and patients suffering from Crohn’s disease, ischemic stroke, GvHD, and myocardial infarction. They reviewed 2347 citations and included 36 studies, of which 8 were randomized control trials (321 participants). Meta-analysis of the results of these eight trials identified a significant association between transient fever and MSCs administration. No significant association was found between immediate events like acute infusional toxicity, organ system complications, infections, and long-term adverse events (death and malignancy).

In a systematic review, Toyserkani et al. [89] also confirmed the safety profile of AD-MSCs therapy. They performed a systematic search to identify adverse events of AD-MSCs application. They particularly focused on the risk of thromboembolic events, and immunological and oncologic safety. They found 70 studies, which involved more than 1400 patients. Only a few cases of thromboembolic complications were found in studies involving systemic or cardiac administration of AD-MSCs cell therapy. Only one case of breast cancer recurrence was identified in 121 patients within a 12-month period, which confirms the oncological safety of the therapy; however; long-term follow-up is necessary. In the case of immunogenicity, 19–34% of patients developed specific antibodies towards donor cells, which means that these cells are not as immune-privileged as they were thought to be. However, the consequences of these reactions are still not defined. However, the authors note that safety assessment was poorly described in the studies and this issue should be taken more carefully and seriously in future studies.

Finally, Wang et al.’s [90] meta-analysis also confirms the safety of MSCs administration in different populations. They searched PubMed, Embase, Web of Science, and Scopus and identified 62 high-quality randomized clinical trials enrolling 3546 patients. They did not find reports of death and infections in selected trials. However, they found a close association between MSCs treatment and transient fever, administration site adverse events, constipation, fatigue, and sleeplessness. These results also confirm the general safety of cell therapies with MSCs.

## 10. The Role of miRNAs in the Modulation of Immune Response

MicroRNAs (miRNAs) are small, non-coding RNAs of 18–22 nucleotides in length. The function of miRNAs is based on maintaining homeostasis of the host immune system. The main mechanism of action involves reducing stability and inhibiting mRNA translation [91]. Commonly, miRNA interacts with the 3′UTR region of the target mRNA and inhibits gene expression. In some cases, it may also interact with the coding region, gene promoter, or 5′UTR fragment. miRNA progresses from the primary miRNA (pri-miRNA), through the precursor miRNA (pre-mRNA) to finally appear as the mature miRNA. The dysfunction of miRNAs contributes to the development of many diseases such as cardiovascular disorders, diabetes, and kidney diseases [92].

The presence of miRNAs in MSCs is essential for their proper proliferation, differentiation, or immunosuppression. The attendance of inflammatory agents in the environment affects the secretion of various factors by MSCs, such as interleukin 6, prostaglandin E2, miR146, and miR155. miRNAs act autologously by affecting the activity of immunomodulatory factors secreted by MSCs or have a paracrine effect on immune cells via extracellular vesicles (EVs) [93].

Studies indicate the possibility of using miRNAs delivered in EV-MSCs, in immune disorders such as allergies, myocardial damage, or Duchenne muscular dystrophy [94]. miR-146a-5p reduced levels of IL-9 and IL-13 and helper T cell cytokines in a group 2 innate lymphoid cell-dominant allergic airway mouse model [95]. In contrast, the use of EVs—umbilical cord blood-derived MSCs in combination with miRNA-181a induced a stronger therapeutic effect after myocardial ischemia-reperfusion injury. MSCs were used to target cells at the site of injury, while miRNA-181a was applied as an immunosuppressive agent. The combination allowed for an anti-inflammatory effect and increased the polarization of regulatory T cells by the c-FOS protein [96].

Furthermore, MSCs inhibit T-cell and B-cell activity, dendritic cell (DC) differentiation, and natural killer (NK) cell proliferation. The miRNAs associated with DC function and maturation include miR-21-5p, miR-142-3p, miR-223-3p, and miR-126-3p. miRNAs presence was confirmed in EVs derived from MSCs. miR-21-5p had the highest expression in the cells studied. Its functions include the degradation of the CCR7 gene, inhibition of IL-12p35, and destruction of IL-6 [97]. BM-MSC-derived EVs, on the contrary, are enriched in miR-125a-3p. It inhibits CD4+ and CD8+ T cells in a mouse model of graft-versus-host disease. Additionally, it improves the viability of CD25+, Foxp3+, and CD4+ regulatory T cells [98]. The dependence between NK cells and miR-122 and miR-15b from EVs- MSCs is also recognized. miRNAs can activate immune cells in a toll-like-receptor (TLR)-dependent manner [99].

Wang et al. [100] described the miRNA expression profile in human AD-MSCs. Seven of the miRNAs studied had different expression levels in the pro-inflammatory cytokine-stimulated group compared to the control group. hsa-miR-543, hsa-miR-155-5p, hsa-miR-146b-5p, and hsa-miR-19b-3p were involved in various immunological processes such as the TGF-beta signaling pathway, FoxO signaling pathway, prolactin signaling pathway or NF-kappa B signaling pathway.

Liu et al. [101] showed that AD-MSCs stimulated with tumor necrosis factor α (TNF α) and lipopolysaccharide (LPS) exhibit memory regarding environmental signals for a short time. In response to treatment with the aforementioned agents, miR155, miR150, and miR 146a remained elevated for at least five days. This suggests that miRNAs are mediators of short-term memory in immune cells as well as MSCs [102].

## 11. Bioengineered AD-MSCs in Regenerative Medicine

Tissue engineering provides new tools to increase the regenerative potential of stem cells obtained from various tissues, including fat tissue. It is currently possible to modify the DNA of stem cells, attach active proteins to their surface, and change their activity by stimulating them with appropriate compounds [78]. In addition, cells can be delivered to tissues on special carriers that increase their pro-regenerative activity [103]. It is worth noting that AD-MSCs are also used in 3D printing (formation of cartilage, fragments of bones, and skin) [104,105]. Modifications of adipose tissue stem cells are aimed, among others, at increasing the proliferative activity of cells, migration, and chemotaxis, increased production of active factors (cytokines, growth factors, exosomes) as well as more effective differentiation into other types of cells, especially osteocytes and chondrocytes [106]. For example, transfected AD-MSCs with miR-27a-3pAD-MSCs had a higher potential to differentiate into osteoblasts, which may be important in the therapy of bone damage and fractures [107]. AD-MSCs are also a good tool for genetic modification. It has recently been shown that using the lentiviral system (vectors expressing Pdx1 and MafA/NeuroD1) it is possible to differentiate them into insulin-producing cells [108]. Using animal models, it has been shown that AD-MSC cells administered on appropriate decellularized intestinal scaffolds (polydopamine (PDA)-mediated surface modification) significantly accelerate intestinal regeneration, stimulate angiogenesis, and inhibit inflammation [109]. In another study, cells were transfected using liposomes with the plasmid Netrin-1. The modified cells showed greater proliferative activity and pro-angiogenic properties. Overexpression of netrin-1 may be a promising strategy for the treatment of ischemic diseases in the future [110].

Cell modification through increased expression of the FGF receptor in another study led to the stimulation of angiogenesis, wound healing, and cell proliferation. In this study, a combination of engineered cells and bacterial cellulose scaffolds enabled the reconstruction of the urethra in an animal model [111].

There are also attempts to modify small extracellular vesicles produced by adipose-derived stem cells. The insertion of the peptide (CREKA) into the vesicle membranes resulted in the retention of the vesicles in the bone and accelerated bone healing [112].

Eke et al. [113] demonstrated that they designed a dermal substitute containing AD-MSCs, that could be used as an improvement in the regeneration of skin, especially on difficult wound beds. AD-MSCs secrete a wide diversity of pro-angiogenic components: fibroblast growth factor 2 (FGF-2), interleukin 6 (IL-6), and vascular endothelial growth factor (VEGF). These factors act at various stages of angiogenesis. Tissue constructs connected with stem cells improve their vascularization. The authors showed that hydrogel created by them had an angiogenic response only with AD-MSCs loaded on them [113].

Using “Click chemistry”, it is also possible to modify the surface of AD-MSCs and increase their ability to penetrate and accumulate in damaged places, e.g., injured liver (homing) [114]. Adding active compounds, such as peptides, antibiotics, and growth factors, to cells creates new possibilities and therapeutic strategies. It can ensure targeted penetration of cells into given tissues and stimulate appropriate biochemical processes in a given place (specific activation of receptors). The “click chemistry” strategy is a powerful tool that has also been recognized by the Nobel Committee (Nobel Prize in Chemistry, 2022) [115].

## 12. Conclusions and Future Perspectives

The abundance of AD-MSCs in fat tissue, the simplicity with which cells can be obtained, their ability to differentiate into multiple lineages, the secretion of various cytokines, and their immunomodulatory effects confirm their essential role in basic science and medicine. AD-MSCs are suitable for testing biological phenomena, drug testing, and regenerative medicine. However, there are still a lot of questions that have not yet been fully answered, inter alia, regarding the effectiveness and safety of therapy in oncological patients. Despite some concerns, AD-MSCs can be used in the treatment of many diseases including skin losses, bone and cartilage defects, and autoimmune diseases. Hence, it seems very important to develop stem-cell-based therapies, especially in university hospitals. It is worth noting that AD-MSC may also be the future in creating the next-generation stem cells. These cells may be the targets for genetic therapy as well as chemical modifications. Modified cells carrying a given drug, e.g., an anti-inflammatory drug, can easily reach hard-to-reach places and act locally without systemic side effects. Cells can also be a carrier of oncolytic viruses and anticancer molecules. This creates completely new tools to fight cancer. However, new applications of cells still require further, detailed pre-clinical and clinical trials.

## Figures and Tables

**Table 1:** Positive and negative markers of AD-MSCs and their function in cells (The information in the table below has been compiled using https://www.ncbi.nlm.nih.gov/gene (accessed on 28 November 2022), search: a particular marker).

Surface Marker	Gene	Name	Function	AD-MSCs Expression
CD10	*MME*	membrane metalloendopeptidase	neutral endopeptidase; inactivates several peptide hormones including glucagon, enkephalins, substance P, neurotensin, oxytocin, and bradykinin; common acute lymphocytic leukemia antigen.	positive
CD105	*ENG*	endoglin	I transmembrane protein—induce activation and proliferation of endothelial cells.	positive
CD13	*ANPEP*	Alanyl aminopeptidase, membrane	takes part in the final digestion of peptides generated from hydrolysis of proteins by gastric and pancreatic protease.	positive
CD26	*DPP4*	dipeptidyl peptidase 4	this protein encoded by this gene is involved in glucose metabolism by N-terminal truncation and inactivation of the incretins glucagon-like peptide-1 (GLP) and gastric inhibitory protein (GIP).	positive
CD29	*ITGB1*	integrin subunit beta 1	cell adhesion.	positive
CD36	*CD36*	CD36 molecule	cell adhesion; receptor for thrombospondin in platelets and various cell lines.	positive
CD44	*CD44*	CD44 molecule	cell adhesion; cell migration; cell-cell interactions.	positive
CD49d	*ITGA4*	integrin subunit alpha 4	cell surface adhesion and signaling.	positive
CD49e	*ITGA5*	integrin subunit alpha 5	cell surface adhesion and signaling; this integrin may promote tumor invasion.	positive
CD59	*CD59*	CD59 molecule	this gene encodes a cell surface glycoprotein—regulates complement-mediated cell lysis; it is involved in lymphocyte signal transduction; inhibitor of the complement membrane attack complex.	positive
CD73	*NT5E*	5′-nucleotidase ecto	plasma membrane protein that catalyzes the conversion of extracellular nucleotides to membrane-permeable nucleosides; determinant of lymphocyte differentiation.	positive
CD90	*THY1*	Thy-1 cell surface antigen	the encoded protein is involved in cell adhesion and cell; communication in particularly in cells of the immune and nervous systems; marker for hematopoietic stem cells.	positive
CD106	*VCAM1*	vascular cell adhesion molecule 1	mediates the adhesion of lymphocytes, monocytes, eosinophils, and basophils to vascular endothelium; involved in leukocyte-endothelial cell signal transduction.	positive
CD11b	*ITGAM*	integrin subunit alpha M	regulates leukocyte adhesion and migration; participates in the processes of the immune system.	negative
CD14	*CD14*	CD14 molecule	surface antigen expressed on monocytes/macrophages; mediate the innate immune response.	negative
CD79A	*CD79A*	CD79a molecule	the B lymphocyte antigen receptor.	negative
CD19	*CD19*	CD19 molecule	expression of this cell surface protein is restricted to B cell lymphocytes; this protein forms a complex with several membrane proteins including complement receptor type 2 (CD21) and tetraspanin (CD81) and this complex reduces the threshold for antigen-initiated B cell activation; activation of this B-cell antigen receptor complex activates the phosphatidylinositol 3-kinase signaling pathway and the subsequent release of intracellular stores of calcium ions.	negative
CD253a	*GYPA*	glycophorin A	major membrane glycoprotein; marker of cells of the erythroid lineage.	negative
CD31	*PECAM1*	platelet and endothelial cell adhesion molecule 1	involved in leukocyte transmigration; angiogenesis; integrin activation.	negative
CD34	*CD34*	CD34 molecule	role in the attachment of stem cells to the bone marrow extracellular matrix or stromal cells.	variable expression
CD45	*PTPRC*	protein tyrosine phosphatase receptor type C	regulate cell growth; regulate differentiation; regulate mitosis; regulate oncogenic transformation; regulator of T- and B-cell antigen receptor signaling; regulator of cytokine receptor signaling—suppresses JAK kinases.	negative
CD49f	*ITGA6*	integrin subunit alpha 6	cell surface adhesion and signaling.	negative
CD56	*NCAM1*	neural cell adhesion molecule 1	cell adhesion.	negative
CD62	*SELP*	selectin P	mediates leukocyte-platelet and leukocyte-vascular endothelial cell adhesion.	negative
HLA-DRA	*HLA-DRA*	major histocompatibility complex, class II, DR alpha	is expressed on the surface of various antigen-presenting cells such as B lymphocytes, dendritic cells, and monocytes/macrophages; plays a central role in the immune system and response by presenting peptides derived from extracellular proteins, in particular, pathogen-derived peptides to T cells.	negative
HLA-DRB1	*HLA-DRB1*	major histocompatibility complex, class II, DR beta 1	responsible for presenting peptides derived from extracellular proteins.	negative
HLA-DRB3	*HLA-DRB3*	major histocompatibility complex, class II, DR beta 3	responsible for presenting peptides derived from extracellular proteins.	negative
HLA-DRB4	*HLA-DRB4*	major histocompatibility complex, class II, DR beta 4	responsible for presenting peptides derived from extracellular proteins.	negative
HLA- DRB5	*HLA-DRB5*	major histocompatibility complex, class II, DR beta 5	responsible for presenting peptides derived from extracellular proteins.	negative
NA	*PODXL*	podocalyxin like	the encoded protein was identified as a component of glomerular podocytes; role in hematopoietic cell differentiation.	negative

**Table 4:** The application of AD-MSCs in selected clinical models.

	Medical Conditions	Source of AD-MSCs	Phase Study	Research Model	NCT Number
Skin diseases	Keloids	Autologous AD-MSCs	Phase 2	8 participants; Lipoaspirate was collected from each patient and stromal vascular fraction was infiltrated into the keloid tissue	NCT04553159
	Burn	Allogenic AD-MSC	Phase 1	5 participants; Patients with wounds resulting from second-degree burns were applied with an ALLO-AD-MSC-DFU dressing containing AD-MSCs	NCT02394873
	Chronic wounds associated with diabetes, venous and pressure ulcers	Autologous AD-MSCs	Phase 2	25 participants; Injection around and within the wound of AD-MSCs obtained from lipoaspirate	NCT02092870
Orthopedic diseases	Knee osteoarthritis	Allogenic AD-MSCs	Phases 1 and 2	57 participants; Administration of allogeneic AD-MSCs in three amounts—1.6 × 10^7^; 3.2 × 10^7^; 6.4 × 10^7^ cells	NCT02784964
	Rotator cuff tear	Allogenic AD-MSCs	Phase 2	24 participants; Patients were administered allogeneic AD-MSCs in scaffolds of fibrin glue	NCT02298023
	Rheumatoid arthritis	Autologous AD-MSCs	Phases 1 and 2	15 participants; Administer a single dose of stem cells as an intravenous infusion	NCT03691909
Cardiovascular diseases	Peripheral arterial disease	Autologous AD-MSCs	Phase 1	10 participants; Single administration of AD-MSC or pretreatment of patients with ultrasound therapy	NCT02756884
	Cardiovascular disorders, including myocardial infarction, atherosclerosis, and coronary artery disease	Autologous AD-MSCs	Phase 1	14 participants; Collection of lipoaspirate followed by administration of adipose-tissue-derived stem cells	NCT00442806
	Heart failure	Allogenic AD-MSCs	Phase 2	138 participants; Direct injection of AD-MSCs from the Cardiology Stem Cell Center into the heart muscle	NCT02673164
Autoimmune diseases	Systemic sclerosis	Autologous stromal vascular fraction (SVF)	Phase 1	20 participants; Injection of SVF obtained from lipoaspirate using automated methods	NCT03060551
	Rheumatoid arthritis	Allogenic AD-MSCs	Phases 1 and 2	53 participants; Intravenous infusion of AD-MSCs with simultaneous treatment with a non-biological drug that modifies the disease	NCT01663116
	Rheumatoid arthritis	Autologous AD-MSCs	Phases 1 and 2	15 participants; A single intravenous infusion of AD-MSCs obtained from patients with rheumatoid arthritis	NCT03691909
Other diseases	Liver cirrhosis	Autologous AD-MSCs	Phase 1	6 participants; Hepatic injection of autologous AD-MSCs in patients with cirrhosis of the liver	NCT02297867
	Chronic obstructive pulmonary disease	Autologous AD-MSCs	Phases 1 and 2	26 participants; Provision of AD-MSC along with SVF obtained by liposuction	NCT02216630
	Crohn’s fistula	Autologous AD-MSCs	Phase 2	40 participants; Injection of AD-MSCs in the form of a preparation called ADIPOPLUS	NCT01011244

## Data Availability

The work does not contain any additional information or other supporting data.
